# The Effectiveness of Internet-Based Self-Help Interventions to Reduce Suicidal Ideation: Protocol for a Systematic Review and Meta-Analysis

**DOI:** 10.2196/14174

**Published:** 2019-07-29

**Authors:** Rebekka Büscher, Michelle Torok, Lasse Sander

**Affiliations:** 1 Department of Rehabilitation Psychology and Psychotherapy University of Freiburg Freiburg Germany; 2 Black Dog Institute University of New South Wales Sydney Australia

**Keywords:** suicide, suicidal ideation, internet, computer-assisted therapy, randomized controlled trial, systematic review, meta-analysis

## Abstract

**Background:**

Suicidal ideation is a highly prevalent condition. There are several barriers for individuals to seek treatment that may be addressed by providing internet-based self-help interventions (ISIs). Current evidence suggests that ISIs for mental disorders may only be effective in reducing suicidal ideation if they specifically target suicidal thoughts or behaviors.

**Objective:**

The aim of this systematic review and meta-analysis is to investigate the effectiveness of ISIs that directly target suicidal thoughts or behaviors.

**Methods:**

We will conduct a sensitive systematic literature search in PsycINFO, MEDLINE, the Cochrane Central Register of Controlled Trials, and the Centre for Research Excellence of Suicide Prevention databases. Only randomized controlled trials evaluating the effectiveness of ISIs for suicide prevention will be included. Interventions must be delivered primarily in a Web-based setting; mobile-based interventions and interventions targeting gatekeepers will be excluded. Suicide ideation will be the primary outcome; secondary outcomes will be completed suicides, suicide attempts, depressiveness, anxiety, and hopelessness. Study quality will be assessed using the Cochrane Risk of Bias tool. We will provide a narrative synthesis of included studies. If studies are sufficiently homogenous, we will conduct a meta-analysis of the effectiveness on suicide ideation and, if possible, we will evaluate publication bias using funnel plots. We will evaluate the cumulative evidence in accordance with the Grading of Recommendations Assessment, Development and Evaluation framework.

**Results:**

This review is in progress, with findings expected by August 2019.

**Conclusions:**

This systematic review and meta-analysis focuses on the effectiveness of ISIs for suicidal thoughts and behaviors. It will provide guidance to clinical practice and encourage further research by synthesizing the best available evidence.

**Trial Registration:**

International Prospective Register of Systematic Reviews (PROSPERO) CRD42019130253; https://www.crd.york.ac.uk/prospero/display_record.php?RecordID=130253

**International Registered Report Identifier (IRRID):**

PRR1-10.2196/14174

## Introduction

### Importance

Suicide is a severe public health problem. Globally, more than 800,000 people die because of suicide each year, and it is the second leading cause of death among those aged 15 to 29 years [1]. Suicide attempts are estimated to be 20 times more prevalent than completed suicides [2]. Globally, suicide rates have increased by 60% within the last 45 years [2].

Although there are effective suicide prevention strategies [3], many people at risk of suicide do not seek treatment, which may limit the effectiveness or impact of these strategies [4]. Surveys conducted worldwide (*N*=55,302 participants) by the World Health Organization show that only 39% of people with suicidal behavior had received any kind of therapeutic intervention for emotional difficulties in the preceding 12 months [5]. Treatment, including treatment of suicidal thoughts, was most prevalent in high-income countries (56% received treatment within the past 12 months) and less frequent in middle-income (28%) and low-income (17%) countries. Common barriers to treatment-seeking have been identified as (1) the wish to solve the problem by oneself, (2) the belief that one would get better without treatment, (3) the belief that the problem was not that severe, (4) stigma, (5) structural problems, for example, financial effort and low availability of treatment, and (6) low perceived need [5].

### Internet-and-Mobile-Based Interventions

Over the past two decades, the development and evaluation of internet-and mobile-based interventions has been an emerging focus of mental health research. Previous meta-analyses have verified the effectiveness of digital interventions for a variety of mental disorders and health issues, including depression [6-10], anxiety [6] and post-traumatic disorder [11]. Internet-based interventions have now been integrated in clinical practice in several countries, including Australia, the Netherlands, Sweden, Norway, and England [12,13], demonstrating their value as a part of health care for at-risk individuals. Within the diverse field of digital interventions, internet-based self-help interventions (ISIs) are the most commonly developed and used. ISIs are stand-alone interventions that provide participants with evidence-based therapeutic material, which can be used self-reliantly [12]. ISIs can involve different levels of human support [14]. In guided ISIs, a clinician accompanies the intervention by providing feedback or guidance on the tasks and progress, often on a weekly basis [14]. Human support is typically limited to positive reinforcement, giving feedback and clarification of content instead of delivering additional therapeutic techniques [12].

The use of ISIs might address several of the barriers mentioned above: (1) The desire to “solve the problem by oneself” can be appropriately addressed by using guided or unguided self-help interventions [12,14]. (2) Individuals who assume that they will get better without treatment might still look for information or social support online [15], and psychoeducational content can be readily delivered through internet-based interventions. It has also been shown that suicidal individuals spend more time online than nonsuicidal users [16-18], which indicates that ISIs may be very appealing to these individuals. (3) The perception of the problem as not that severe can be addressed by providing a low-threshold program within a stepped-care approach. As a first step, ISIs can be offered, and if the patient does not respond, further programs with more intensive therapeutic support can be provided [12]. (4) Individuals who face stigmatization might benefit from the anonymity that ISIs offer [12], (5) whereas structural barriers can be addressed by the accessibility and flexibility of ISIs. In addition, ISIs can be provided at low costs [19,20]. (6) Although offering self-help interventions does not address the barrier of low perceived need, online self-screening programs may increase the perceived need for treatment by providing feedback to participants [21]. In sum, ISIs may be an appropriate, low-threshold intervention for individuals at risk of suicide.

### State of Research: Internet-Based Self-Help Interventions for Suicide Prevention

ISIs for suicide prevention have been developed in recent years. In response to the growth of electronic health (eHealth) interventions in mental health, several reviews and meta-analyses have been published which summarize the evidence of internet-and-mobile-based interventions for suicidality [21-25]. The umbrella term *internet-and- mobile-based suicide prevention* comprises widely divergent approaches, including social networking sites, videos, podcasts, email support programs, mobile apps, gaming interventions [26], self-screenings, text analyses [21], and self-help interventions based on psychological treatment approaches [12]. As a result, reviews have included a variety of interventions.

Overall, those reviews found mixed results and pointed out a paucity of high-quality evidence for the effectiveness of the reviewed interventions. Some reviews reported promising effects of ISIs on suicide-related outcomes [22,23], and one review concluded that there is no evidence for their effectiveness [25]. The most recent of these meta-analyses, by Witt et al [24], found that internet-and-mobile-based interventions (mainly developed for depression treatment) reduced suicide ideation at postintervention. Christensen et al [21] reported that self-help interventions for depression have specific effects on depressiveness but not on suicidal ideation, as there seem to be reductions in suicidal ideation in both depression and control conditions. They included only 1 study with an intervention directly addressing suicidality. It showed a significant effect on suicidal thoughts compared with the control group. The authors concluded that online programs that directly target suicidal ideation and behavior might be more effective than those programs that are designed for mental health more broadly. This is in line with the meta-analytic finding that interventions directly addressing suicidal thoughts and behavior show immediate post-treatment and long-term effects, whereas programs that solely address associated symptoms seem to be only effective in the long term [27].

However, there are some limitations concerning previous reviews in this field. First, ISIs were often not differentiated from other Web-based strategies or mobile-based interventions [22-24]. Differential effects are highly plausible owing to variations in treatment approaches, treatment dose, and application context. Second, several reviews did not restrict study inclusion to controlled trials [21-24] and, third, did not assess risk of bias [21,23]. Fourth, none of the reviews assessed publication bias [21-23,25]. Finally, most reviews did not differentiate between studies investigating interventions that directly targeted suicide versus those that focused on other conditions such as depression or anxiety [22,24,25].

### Objectives

Therefore, this review and meta-analysis will (1) focus on the effectiveness of ISIs directly targeting suicidal thoughts or behavior, (2) exclude Web-based interventions for other health conditions and mobile interventions, and (3) only include randomized controlled trials (RCTs), (4) perform the Cochrane Risk of Bias tool, (5) check for publication bias and (6) search a clinical trial register to give an overview of ongoing trials. As Web-based suicide prevention is a fast-growing field, this review will provide readers with a valuable up-to-date overview of the current state of research and identify gaps in the literature to benefit the design of future research investigations.

## Methods

The review will be reported according to the Preferred Reporting Items for Systematic Reviews and Meta-Analyses (PRISMA) guidelines [28]. This protocol adheres to the PRISMA Protocols [29]. We registered the study with the International Prospective Register of Systematic Reviews (trial registration number: CRD42019130253). Protocol amendments will be tracked and reported in the final publication.

### Eligibility Criteria

#### Population

There will be no restrictions for age groups, gender, or any other sociodemographic variables.

#### Interventions

We will include self-help interventions. They must be delivered predominantly in an online setting, defined as internet-based, online, Web-based, or any other equivalent. Although they are defined as stand-alone internet-based interventions, they may involve some additional human support (eg, guided interventions with written feedback). Interventions that use online tools as an adjunct to face-to-face therapy (eg, blended treatment) will be excluded. Treatment groups must receive a psychological intervention. According to the definition by Kampling et al [30], psychological interventions may comprise elements of cognitive behavioral therapy, psychodynamic psychotherapy, behavior therapy or behavior modification, systemic therapy, third wave cognitive behavioral therapies (eg, dialectical behavior therapy or acceptance and commitment therapy), humanistic therapies, integrative therapies (eg, interpersonal therapy) or other psychological-oriented therapies. The intervention must specifically target suicidal thoughts or behaviors. Interventions that only address symptoms associated with suicidality, for example, depressiveness or anxiety, will not be included. We will include universal, selective, and indicated prevention measures.

#### Comparators

The control group may receive treatment as usual, receive another active or passive treatment, receive placebo, consist of a waiting list group, or receive no intervention. However, controlled trials will not be pooled with comparative trials.

#### Outcomes

Studies will be included if they report a suicide-specific outcome, that is, suicide ideation, suicidal thoughts, or suicidal behaviors (completed suicide or suicide attempts). Suicide attempt is defined as self-injury with the intention to die, in contrast to nonsuicidal self-injury [31]. Outcomes have to be assessed quantitatively. Suicide ideation will be the primary outcome. The following variables will be included in the analyses as secondary outcomes: suicide and suicide attempt, depressiveness, anxiety, and hopelessness. If multiple measures are used, we will prioritize data extraction as follows: (1) validated questionnaires (eg, Beck Scale for Suicide Ideation), (2) clinician ratings, and (3) single item analysis of other rating scales (eg, Patient Health Questionnaire-9 [32]).

#### Study Design

Only published RCTs that are available in full text will be included. The articles have to be provided in English or German language.

#### Exclusion Criteria

Studies will be excluded if the intervention is exclusively mobile based (delivered via a mobile app). Interventions focusing on gatekeepers, for example, health care providers and teachers, will also be excluded. We will not restrict inclusion by year of publication.

### Information Sources and Search Strategy

The systematic literature search will be conducted in the following databases: PsycINFO, MEDLINE, Cochrane Central Register of Controlled Trials (CENTRAL) and the Centre for Research Excellence of Suicide Prevention (CRESP) Database. Search strings enabling a sensitive search (incorporating numerous Medical Subject Headings, subject terms, keywords, and publication types associated with internet, eHealth, suicide, or RCT) were developed for PsycINFO, MEDLINE, and CENTRAL (see Multimedia Appendix 1). As CRESP contains a manageable number of trials, we will screen all studies included in the database. We performed a pilot testing of the outlined search strategy. Hand searches identified 5 eligible trials [33-37]; 100% of these trials were identified by searching the databases using the search strings. In addition, a search in the clinical trial register, ClinicalTrials.gov (provided by the US National Library), will be performed to identify ongoing trials. Hence, this review will provide not only the current state but also the outline of emerging developments in the field. We will screen the reference lists of all included studies and relevant reviews articles for additional studies (backward search), and we will screen studies that cited the included studies and relevant reviews (forward search). In addition, we will perform hand searches. We plan to conduct the searches until April 30, 2019. Gray literature will not be included. Registered trials that have not been published will be used to evaluate possible publication bias.

If it remains unclear whether a study meets the eligibility criteria or if relevant data or analyses have not been reported, we will contact the authors for clarification. We will also contact authors to ask for unpublished results when study protocols without a subsequent publication are identified.

### Data Collection and Analysis

#### Study Records

A total of 2 reviewers (RB and MT) will independently screen the studies for eligibility in a hierarchical approach. The identified articles will be managed in CITAVI. In a first step, the reviewers will screen titles and abstracts identified in the databases. In a second step, they will screen full-text articles. Studies that do not meet the eligibility criteria will be moved to an exclusion folder. Potential discrepancies will be resolved in a discussion with a third researcher (LS). The selection process will be displayed in a PRISMA flowchart [28].

#### Data Extraction and Management

The following information will be extracted from the included studies: study identification items, study design, description of the intervention and control condition, technical characteristics, population, setting, treatment engagement/dropout, outcome variables, and results. We will use a data extraction form. All data will be double-checked by the second reviewer.

#### Assessment of Risk of Bias in Individual Studies

The risk of bias will be assessed with the Cochrane Risk of Bias tool [38] by 2 independent researchers (RB and MT). Potential discrepancies will be resolved in a discussion with a third researcher (LS). The following domains will be analyzed: (a) random sequence generation, (b) allocation concealment, (c) blinding of participants and personnel, (d) blinding of outcome assessment, (e) incomplete outcome data, (f) selective reporting and (g) other sources of bias.

In psychological interventions, blinding of participants or clinicians is not possible. This will result in a high risk of bias rating of (c). We will discuss findings in terms of risk of bias.

#### Qualitative Synthesis

We will narratively describe the relevant characteristics of included interventions and possible limitations of study designs. The relevant results will be reported in text as well as in a summary of findings table in line with the PRISMA guidelines [28].

#### Meta-Analysis

Only studies that provide a quantitative measure of suicide ideation will be included in the meta-analysis. We will analyze heterogeneity by providing *I*^2^ statistics and, if possible, forest plots. According to the GRADE handbook, *I*^2^ <40% indicates low, 30% to 60% indicates moderate, 50% to 90% indicates substantial, and 75% to 100% indicates considerable heterogeneity [39]. If studies fail to show sufficient homogeneity (*I*^2^ <60%) in at least two trials [40], we will not undertake meta-analytic pooling. However, inconsistency may arise from differences in populations, interventions, outcomes, and study methods [39]. If appropriate, we will conduct subgroup analyses according to these categories. We will perform subgroup analyses for adults versus youth, guided versus unguided interventions, and varying control conditions, if possible. A random effects model will be applied. We will estimate standardized mean difference values and the respective 95% CIs. The RevMan software (Review Manager version 5.3 for Windows from the Nordic Cochrane Centre, The Cochrane Collaboration, 2014) will be used for calculation. If possible, sensitivity analyses will be conducted to examine the influence of trials with high risk of bias on the pooled effect size. If meta-analytic pooling is not appropriate, we will only describe reported data narratively.

#### Meta-biases: Confidence in Cumulative Evidence

Trial registrations and study protocols will be identified. This will enable us to determine whether a publication bias is likely, that is, if studies have been published selectively. If the number of retrieved studies is sufficient, we will use visual inspection of funnel plots to assess publication bias and inspect an international trial registry for unpublished studies.

The quality of evidence will be evaluated according to the Grading of Recommendations Assessment, Development and Evaluation (GRADE) [41] by 2 independent researchers (RB and MT). Discrepancies will be resolved in a discussion with a third researcher (LS). Dimensions of the GRADE rating will be risk of bias, inconsistency of results, indirectness of evidence, imprecision of effect size, and publication bias.

## Results

This review is currently in progress. Data extraction started in April 2019. Our final paper is expected to be submitted in September 2019.

## Discussion

Suicide ideation is a highly prevalent condition. Owing to low treatment-seeking [5], it is of great importance to provide individuals at risk of suicide with appropriate and low-threshold treatment options. This systematic review and meta-analysis will address a gap in research by evaluating the effectiveness of ISIs that are specifically designed for suicide prevention. This will provide crucial information for the implementation of ISIs into clinical practice. Hence, we will be able to provide recommendations to policy and research based on the current best available evidence.

## Appendix

Multimedia Appendix 1 Search strings for MEDLINE, PsycINFO and CENTRAL.

## Abbreviations

CENTRAL: Cochrane Central Register of Controlled Trials
CRESP: Centre for Research Excellence of Suicide Prevention
eHealth: electronic health
GRADE: Grading of Recommendations Assessment, Development and Evaluation
ISI: internet-based self-help intervention
PRISMA: Preferred Reporting Items for Systematic Reviews and Meta-Analyses
RCT: randomized controlled trial
