# Supplementary material for: The Effectiveness of Internet-Based Self-Help Interventions to Reduce Suicidal Ideation: Protocol for a Systematic Review and Meta-Analysis
Source: JMIR Res Protoc. 2019 Jul 29;8(7):e14174. doi: 10.2196/14174 (PMC6690160; doi:10.2196/14174)
Supplement: Multimedia Appendix 1 [file resprot_v8i7e14174_app1.pdf]

Multimedia Appendix 1: Search strings for MEDLINE, PsycINFO and CENTRAL

	**MEDLINE via Ebsco**		**PsycINFO via Ebsco**		**CENTRAL[a]**
S1	MH computers	S1	MA computers	S1	MeSH descriptor: [computers] explode all trees
S2	MH software	S2	MA software	S2	MeSH descriptor: [software] explode all trees
S3	MH internet	S3	MA internet	S3	MeSH descriptor: [internet] explode all trees
S4	MH web browser	S4	MA web browser	S4	MeSH descriptor: [web browser] explode all trees
S5	MH technology	S5	MA technology	S5	MeSH descriptor: [technology] explode all trees
S6	TI technolog* OR AB technolog*	S6	TI technolog* OR AB technolog*	S6	(technolog*):ti,ab,kw
S7	software	S7	software	S7	(software):ti,ab,kw
S8	web	S8	web	S8	(web):ti,ab,kw
S9	app	S9	app	S9	(app):ti,ab,kw
S10	internet	S10	internet	S10	(internet):ti,ab,kw
S11	online	S11	online	S11	(online):ti,ab,kw
S12	computer*	S12	computer*	S12	(computer):ti,ab,kw
S13	cyber	S13	cyber	S13	(cyber):ti,ab,kw
S14	TI electronic OR AB electronic	S14	TI electronic OR AB electronic	S14	(electronic):ti,ab,kw
S15	"world wide web" OR www	S15	"world wide web" OR www	S15	("world wide web" OR www):ti,ab,kw
S16	net	S16	net	S16	(net):ti,ab,kw
S17	digital	S17	digital	S17	(digital):ti,ab,kw
S18	virtual	S18	virtual	S18	(virtual):ti,ab,kw
S19	website	S19	website	S19	(website):ti,ab,kw
S20	chat	S20	chat	S20	(chat):ti,ab,kw
S21	forum	S21	forum	S21	(forum):ti,ab,kw
		S22	SU computers		
		S23	SU internet		

		S24	SU information technology		
S22	S1 OR S2 OR S3 OR S4 OR S5 OR S6 OR S7 OR S8 OR S9 OR S10 OR S11 OR S12 OR S13 OR S14 OR S15 OR S16 OR S17 OR S18 OR S19 OR S20 OR S21	S25	S1 OR S2 OR S3 OR S4 OR S5 OR S6 OR S7 OR S8 OR S9 OR S10 OR S11 OR S12 OR S13 OR S14 OR S15 OR S16 OR S17 OR S18 OR S19 OR S20 OR S21 OR S22 OR S23 OR S24	S22	S1 OR S2 OR S3 OR S4 OR S5 OR S6 OR S7 OR S8 OR S9 OR S10 OR S11 OR S12 OR S13 OR S14 OR S15 OR S16 OR S17 OR S18 OR S19 OR S20 OR S21
S23	MH computer-assisted instruction	S26	MA computer-assisted instruction	S23	MeSH descriptor: [computer-assisted instruction] explode all trees
S24	MH therapy, computer-assisted	S27	MA therapy, computer-assisted	S24	MeSH descriptor: [therapy, computer-assisted] explode all trees
S25	MH medical informatics	S28	MA medical informatics	S25	MeSH descriptor: [medical informatics] explode all trees
S26	MH distance counseling	S29	MA distance counseling	S26	MeSH descriptor: [distance counseling] explode all trees
S27	e-therap*	S30	e-therap*	S27	(e-therap*):ti,ab,kw
S28	"e-mental health" OR "emental health"	S31	"e-mental health" OR "emental health"	S28	("e-mental health" OR "emental health"):ti,ab,kw
S29	e-health OR ehealth	S32	e-health OR ehealth	S29	(e-health OR ehealth):ti,ab,kw
S30	"electronic health"	S33	"electronic health"	S30	("electronic health"):ti,ab,kw
S31	telecare	S34	telecare	S31	(telecare):ti,ab,kw
S32	tele-health OR telehealth	S35	tele-health OR telehealth	S32	(tele-health OR telehealth):ti,ab,kw
S33	tele-medicine OR telemedicine	S36	tele-medicine OR telemedicine	S33	(tele-medicine OR telemedicine):ti,ab,kw
S34	tele-rehabilitation OR telerehabilitation	S37	tele-rehabilitation OR telerehabilitation	S34	(tele-rehabilitation OR telerehabilitation):ti,ab,kw
S35	iCBT OR i-CBT	S38	iCBT OR i-CBT	S35	(iCBT OR i-

					CBT):ti,ab,kw
S36	cCBT OR c-CBT	S39	cCBT OR c-CBT	S36	(cCBT OR c-CBT):ti,ab,kw
S37	"personal digital assist*" OR PDA	S40	"personal digital assist*" OR PDA	S37	("personal digital assist*" OR PDA):ti,ab,kw
S38	"patient monitor*"	S41	"patient monitor*"	S38	("patient monitor*):ti,ab,kw
		S42	SU internet-assisted instruction		
		S43	SU telemedicine		
		S44	SU online therapy		
		S45	SU computer assisted therapy		
S39	S23 OR S24 OR S25 OR S26 OR S27 OR S28 OR S29 OR S30 OR S31 OR S32 OR S33 OR S34 OR S35 OR S36 OR S37 OR S38	S46	S26 OR 27 S28 OR S29 OR S30 OR S31 OR S32 OR S33 OR S34 OR S35 OR S36 OR S37 OR S38 OR S39 OR S40 OR S41 OR S42 OR S43 OR S44 OR S45	S39	S23 OR S24 OR S25 OR S26 OR S27 OR S28 OR S29 OR S30 OR S31 OR S32 OR S33 OR S34 OR S35 OR S36 OR S37 OR S38
S40	MH suicide	S47	MA suicide	S40	MeSH descriptor: [suicide] explode all trees
S41	MH self-injurious behavior	S48	MA self-injurious behavior	S41	MeSH descriptor: [self-injurious behavior] explode all trees
S42	MH suicidal ideation	S49	MA suicidal ideation	S42	MeSH descriptor: [suicidal ideation] explode all trees
S43	MH suicide, attempted	S50	MA suicide, attempted	S43	MeSH descriptor: [suicide, attempted] explode all trees
S44	suicid*	S51	suicid*	S44	(suicid*)
S45	self-injur*	S52	self-injur*	S45	(self-injur*):ti,ab,kw
S46	self-harm OR selfharm	S53	self-harm OR selfharm	S46	(self-harm OR selfharm):ti,ab,kw
S47	self-mutilation OR selfmutilation	S54	self-mutilation OR selfmutilation	S47	(self-mutilation OR selfmutilation):ti,ab,kw
S48	auto-mutilation OR	S55	auto-mutilation OR	S48	(auto-mutilation OR

	automutilation		automutilation		automutilation):ti,ab, kw
		S56	SU suicide		
		S57	SU suicidal ideation		
		S58	SU attempted suicide		
		S59	SU suicidology		
		S60	SU self-injurious behavior		
		S61	SU self-mutilation		
		S62	SU suicide prevention		
S49	S40 OR S41 OR S42 OR S43 OR S44 OR S45 OR S46 OR S47 OR S48	S63	S47 OR S48 OR S49 OR S50 OR S51 OR S52 OR S53 OR S54 OR S55 OR S56 OR S57 OR S58 OR S59 OR S60 OR S61 OR S62	S49	S40 OR S41 OR S42 OR S43 OR S44 OR S45 OR S46 OR S47 OR S48
S50	MH randomized controlled trials as topic	S64	MA randomized controlled trials as topic		
S51	MH clinical trials as topic	S65	MA clinical trials as topic		
S52	PT randomized controlled trial	S66	PT randomized controlled trial		
S53	PT controlled clinical trial	S67	PT controlled clinical trial		
S54	PT clinical trial	S68	PT clinical trial		
S55	PT clinical trial protocol	S69	PT clinical trial protocol		
S56	PT clinical study	S70	PT clinical study		
S57	RCT	S71	RCT		
S58	clinical trial	S72	clinical trial		
S59	random*	S73	random*		
S60	TI trial OR AB trial	S74	TI trial OR AB trial		
S61	S50 OR S51 OR S52 OR S53 OR S54 OR S55 OR S56 OR S57 OR S58 OR S59 OR S60	S75	S64 OR S65 OR S66 OR S67 OR S68 OR S69 OR S70 OR S71 OR S72 OR S73 OR S74		
S62	S22 OR S39	S76	S25 OR S46	S50	S22 OR S39

S63	S62 AND S49 AND S61	S77	S76 AND S63 AND S75	S51	S50 AND S49

Note. MH/MA: MeSH Term; TI: title; AB: abstract; SU: subject terms; PT: publication type ti,ab,kw: title, abstract, keywords.

[a]As the CENTRAL database only includes controlled trials, the search string for CENTRAL does not comprise search terms related to study design.
